# In Silico Novel Drug Design Targeting the Oral Microbiome: Endodontic and Periodontal Pathogenic Bacteria

**DOI:** 10.3390/microorganisms9112400

**Published:** 2021-11-22

**Authors:** Maurilio D’Angelo, Alessio Zanza, Luca Testarelli, Federico Valenti Obino, Andrea Cicconetti

**Affiliations:** Department of Oral and Maxillo Facial Sciences, University of Rome La Sapienza, 00161 Rome, Italy; maurilio.dangelo@uniroma1.it (M.D.); ale.zanza@gmail.com (A.Z.); Federico.valenti@uniroma1.it (F.V.O.); andrea.cicconetti@uniroma1.it (A.C.)

The oral cavity is composed of about 300 million species of bacteria that occupy various ecological niches. It is well known that dental plaque is a major ecological niche and is a biofilm composed of diverse bacterial species housed in a exopolysaccharide matrix.

Additionally, it is important to remember that many bacterial species are found in saliva, gingival crevice fluid, and colonizing soft tissues such as the back of the tongue and tonsils. The different compositions of these fluids, depending on their constituents, can significantly change the number of bacteria present and their ability to replicate [1].

The concepts of Microbiome, Microbiota and Quorum Sensing have now allowed for a profound understanding of how bacteria are influenced by the external environment and by the presence or proximity of bacteria of other species or of the same species [2].

This evaluation, related to how the endogenous molecules present in fluids and exudates modify bacterial concentration and replication, offers an interesting starting point for future research: how do endogenous molecules act on the bacterial population of a certain district?

Subsequently, better understanding the mechanisms underlying the modifications of endogenous secretions and endogenous molecules is necessary, with increasing attention on psycho–neuro–immuno–endocrine correlations [1,3,4,5].

From this point of view, the importance of sequencing bacterial genomes becomes fundamental to better understanding the modifications that bacteria have according to the environment in which they are found how they manage to modify their gene expression in relation to the environment, and vice versa, how human tissues respond to the presence of different bacterial species and the different activities they can manifest [6,7,8].

Considering the rich presence of fluids in periodontal tissues and how they offer substrates and nutrients to bacteria, it is of fundamental importance to understand these aspects and to evaluate the possibility of conveying molecules and drugs that can improve tissue health through these fluids [1,5,9]. The evaluation of the characteristics of bacteria that cause endodontic alterations is also fundamental [10]. Conducting a study of the bacterial populations present makes it possible to devise personalized approaches to endodontic treatment and, in the near future, with a microbiogram-guided approach [11,12].

In this context, it should also be emphasized that many oral diseases have a microbial etiology.

Often, the individual predispositions of patients favor the possible pathogenicity of some bacterial species, which in other conditions turn out to be commensal bacteria with reduced pathogenic capacities. As a function of this, more and more studies aim to evaluate how certain hormonal concentrations affect bacterial pathogenicity [13,14].

What makes these approaches interesting is how all of these factors influence tissue inflammation levels and inflammatory mediator molecules and how these are altered, in combination with immunity cells, the bacterial population, their genetic expression, and hence their pathogenicity, in reciprocal action [15].

More and more studies will be directed at understanding the reciprocal modifications between bacterial population and human tissues and how inflammation of periodontal and peri-implant tissues, also as a function of the different surfaces, alters this delicate balance, fundamental for oral health, and the maintenance of dental therapies.
